# Supplementary material for: Characterisation of Cell-Mediated Immunity Against Bovine Alphaherpesvirus 1 (BoAHV-1) in Calves
Source: Vaccines (Basel). 2025 Sep 23;13(10):996. doi: 10.3390/vaccines13100996 (PMC12567685; doi:10.3390/vaccines13100996)
Supplement: Supplementary file 1 [file vaccines-13-00996-s001.zip › vaccines-3805167-supplementary.pdf]

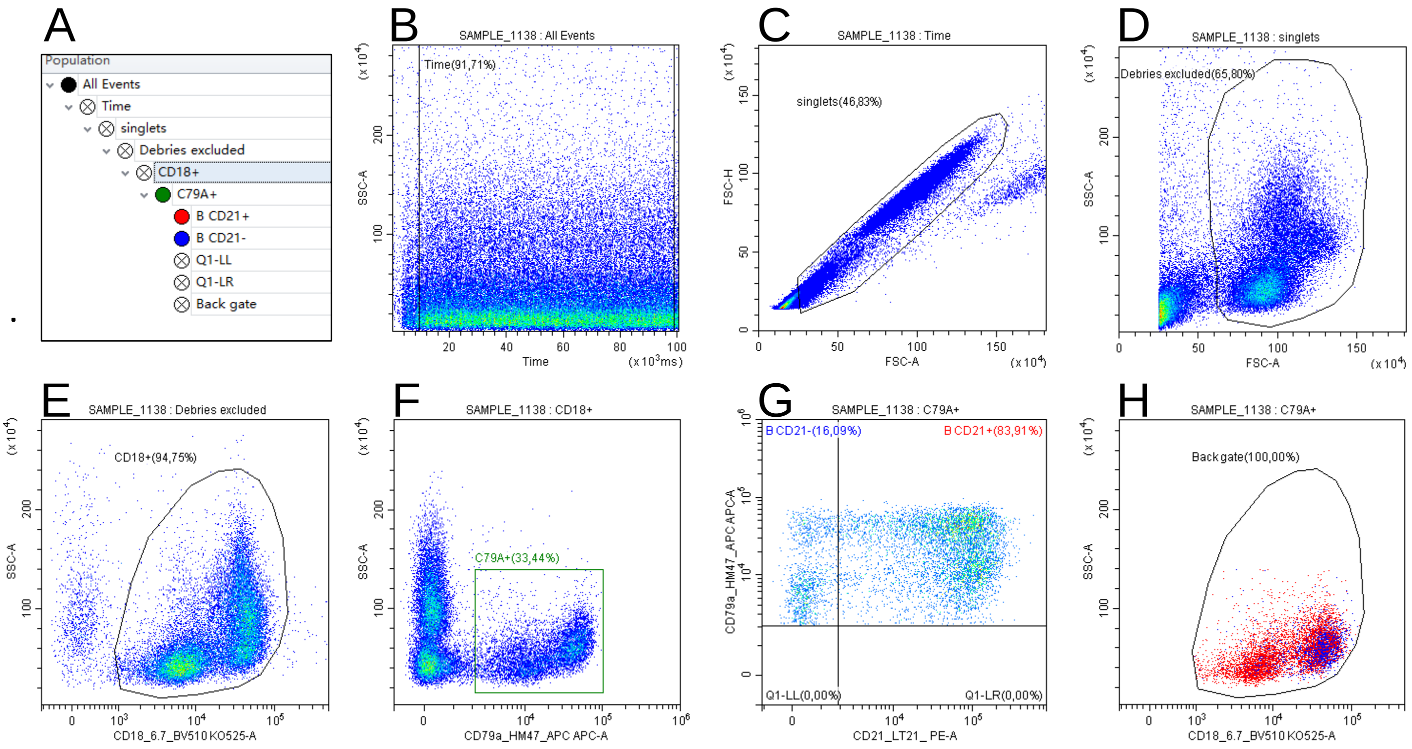

Supplementary Figure S1. Gating strategy used to analyze B lymphocyte subsets.

Boxes (A–E) show the same initial gating steps described in Figure 2. (F) All B lymphocytes were identified in the CD79a vs SSC-A dot plot as “CD79a+” cells. This gate was then applied in the CD21 vs CD79a dot plot (G) to quantify the percentages of CD21+ (red) and CD21− (blue) subsets. (H) Back-gating analysis in the CD18 vs SSC-A dot plot confirmed the upstream gating strategy and showed the different levels of CD18 expression between these two subsets.

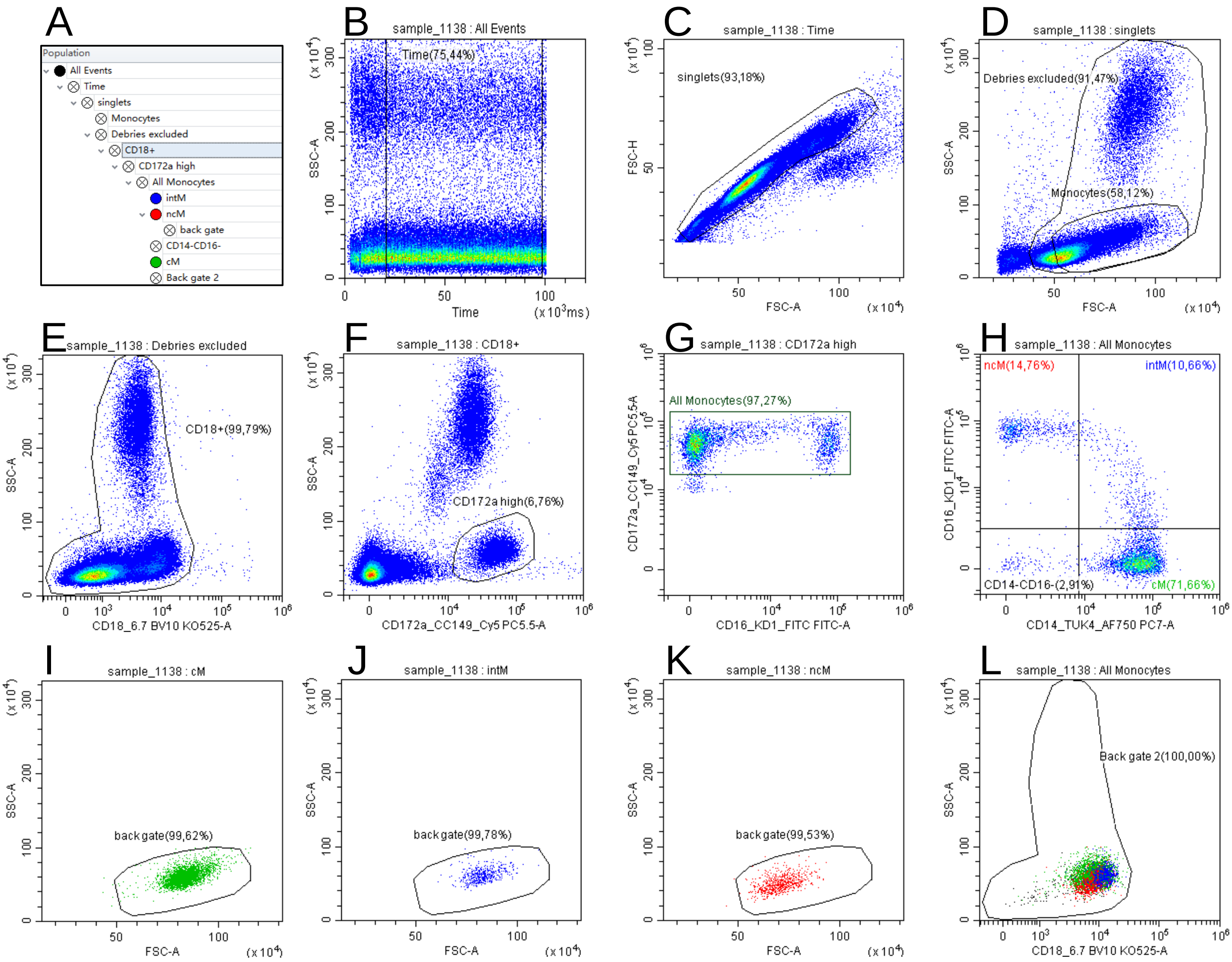

Spplementary Figure S2. Gating strategy used to analyze monocyte subsets.

Boxes (A–E) show the same initial gating steps described in Figure 2. (F) The "CD18+" gate was applied to the CD172a vs SSC-A dot plot to identify the "CD172 high" cells. This gate was then used in the CD16 vs CD172a dot plot (G) to identify "all monocytes", according to Grandoni et al. (2023). (H) Finally, this gate was used in the CD14 vs CD16 dot plot to identify the cM (green), intM (blue), and ncM (red) subsets. (I–K) Back-gating analysis confirmed that these final gated populations originated within the expected region according Grandoni et al. (2021) in the initial FSC-A vs SSC-A dot plot, validating the upstream gating strategy. (L) Additional back-gating was performed in the CD18 vs SSC-A dot plot, highlighting the different levels of CD18 expression among these subsets.

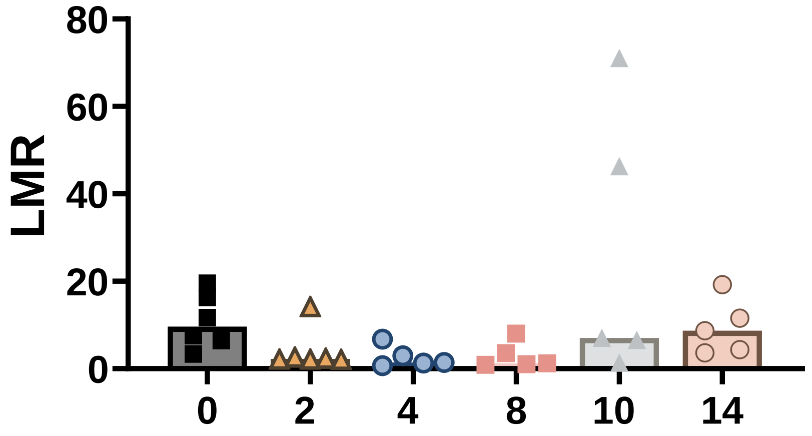

Supplementary Figure S3. Lymphocyte to monocyte ratio in calves infected infected with *BoAHV-1*. Six calves were infected with wild-type BoAHV-1by intranasal route. EDTA blood samples were collected before infection (day 0) and post-infection (2, 4, 8, 10, 14 dpi). Changes in the levels of circulating lymphocytes and monocytes levels were monitored with complete blood count and then lymphocyte to monocyte ratio (LMR) were calcuated.
